# Social media as a recruitment platform for a nationwide online survey of COVID-19 knowledge, beliefs, and practices in the United States: methodology and feasibility analysis

**DOI:** 10.1186/s12874-020-01011-0

**Published:** 2020-05-13

**Authors:** Shahmir H. Ali, Joshua Foreman, Ariadna Capasso, Abbey M. Jones, Yesim Tozan, Ralph J. DiClemente

**Affiliations:** 1 Department of Social and Behavioral Sciences, School of Global Public Health, New York University, 715 Broadway, New York, NY 10003 USA; 2 Ophthalmology, Department of Surgery, University of Melbourne, Melbourne, Australia; 3 Department of Epidemiology, School of Global Public Health, New York University, New York, USA; 4 Global Health Program, School of Global Public Health, New York University, New York, USA

**Keywords:** COVID-19, Coronavirus, Knowledge, Beliefs, Practices, Infectious disease, Social media, Facebook, Surveillance, Methodology

## Abstract

**Background:**

The COVID-19 pandemic has evolved into one of the most impactful health crises in modern history, compelling researchers to explore innovative ways to efficiently collect public health data in a timely manner. Social media platforms have been explored as a research recruitment tool in other settings; however, their feasibility for collecting representative survey data during infectious disease epidemics remain unexplored.

**Objectives:**

This study has two aims 1) describe the methodology used to recruit a nationwide sample of adults residing in the United States (U.S.) to participate in a survey on COVID-19 knowledge, beliefs, and practices, and 2) outline the preliminary findings related to recruitment, challenges using social media as a recruitment platform, and strategies used to address these challenges.

**Methods:**

An original web-based survey informed by evidence from past literature and validated scales was developed. A Facebook advertisement campaign was used to disseminate the link to an online Qualtrics survey between March 20–30, 2020. Two supplementary male-only and racial minority- targeted advertisements were created on the sixth and tenth day of recruitment, respectively, to address issues of disproportionate female- and White-oriented gender- and ethnic-skewing observed in the advertisement’s reach and response trends.

**Results:**

In total, 6602 participant responses were recorded with representation from all U.S. 50 states, the District of Columbia, and Puerto Rico. The advertisements cumulatively reached 236,017 individuals and resulted in 9609 clicks (4.07% reach). Total cost of the advertisement was $906, resulting in costs of $0.09 per click and $0.18 per full response (completed surveys). Implementation of the male-only advertisement improved the cumulative percentage of male respondents from approximately 20 to 40%.

**Conclusions:**

The social media advertisement campaign was an effective and efficient strategy to collect large scale, nationwide data on COVID-19 within a short time period. Although the proportion of men who completed the survey was lower than those who didn’t, interventions to increase male responses and enhance representativeness were successful. These findings can inform future research on the use of social media recruitment for the rapid collection of survey data related to rapidly evolving health crises, such as COVID-19.

## Background

COVID-19, the disease caused by the novel coronavirus SARS-CoV-2, was first detected in Wuhan, China, in December 2019, and has since evolved into a global pandemic in 2020, producing one of the largest global health crises in modern history. Along with pressing requirements for surveillance data on COVID-19 incidence, morbidity, and mortality, it is essential to effectively and efficiently collect data to gauge population-level experiences related to COVID-19. Specifically, given that much of the prevention and societal impacts of the COVID-19 pandemic are driven by behavioral decisions, such as hygiene practices and social distancing [1], collecting insights on knowledge, beliefs, and practices related to COVID-19 can be valuable in informing public health professionals and policy makers developing targeted interventions and effective behavior change communication campaigns.

With the emergence of the COVID-19 outbreak, studies have utilized web-based participant recruitment platforms [2] or informal social media campaigns [3] to collect national data describing COVID-19-related knowledge, perceptions, and behaviors. The use of formal advertisement-based social media recruitment campaigns (such as Facebook advertisements) to rapidly obtain representative survey data has shown some promise in other research contexts [4, 5]; however, it remains largely unexplored for online survey data collection during infectious disease outbreaks [6–8]. According to the Pew Research Center, in 2019, Facebook was the second most popular social media platform, used by 69% of U.S. adults [9]. In addition to its popularity, an advantage of Facebook over other online platforms is that 74% of adult users access it on a daily basis, and it is the social platform most frequently accessed by all age groups, including older adults [10].

The aim of this study was to analyze preliminary recruitment and participant data from a nationwide COVID-19 knowledge, beliefs, and practices survey conducted using a Facebook advertising campaign. Specifically, we provide an in-depth discussion of the methodology used to design and implement the campaign, the measures taken to address the challenges encountered, and an overview of the characteristics of the recruited sample. In doing so, we hope to inform future work attempting to use social media-based recruitment for research on the COVID-19 pandemic or similar public health crises.

## Methods

### Questionnaire development

The target population of the survey was adults aged 18 years or older currently residing in the United States (U.S.). An original survey was developed by study authors informed by three sources: 1) the Health Belief Model, a widely used model for explaining and predicting health behaviors [11]; 2) empirically validated scales with established psychometrics; and 3) in-depth review of past survey research focused on knowledge, beliefs, and practices pertaining to infectious disease epidemics, including H1N1 [12, 13], MERS [14], SARS [6, 15, 16], and Ebola [17, 18].

The full survey (Supplemental File 1) included 119 unique question items and was administered via Qualtrics (Provo, UT). Survey items collected data on a range of relevant topics, including: 1) knowledge of COVID-19; 2) knowledge of COVID-19 prevention measures; 3) sources of COVID-19 knowledge; 4) COVID-19 prevention and emergency preparedness behaviors; 5) COVID-19 beliefs, attitudes, and risk perceptions; 6) attitudes towards select COVID-19 policies; 7) COVID-19-induced anxiety and depression using an adapted version of the 4-item Patient Health Questionnaire [19, 20] and; 8) COVID-19 subjective impact using an adapted version of the 6-item Impact of Event Scale [16, 21]. Participants provided information on sex, age, race, state of residence (50 states plus Washington D.C. and Puerto Rico), type of residence (urban, suburban, rural), employment, presence of children in the household, education, and political affiliation. U.S. states were further categorized by geographical region according to the U.S. Bureau of the Census categories [22]. Due to the significant amount of demographics questions, to minimize survey fatigue, the majority these questions were placed at the end of the survey with the exception of age and sex. The estimated completion time of the survey was 10–15 min based on pilot testing conducted among the study team members.

Before implementing the survey, participants were asked two eligibility screening questions to determine their age and country of residence. Those who responded that they were below 18 years of age or who did not reside in the United States were not eligible to participate, and they received a “thank you” message with links to the Centers for Disease Control and Prevention and World Health Organization websites for further information on COVID-19. Partially completed surveys were automatically recorded if no activity was observed for 24 h. Eligible participants who completed the survey were directed to a final “thank you” page with links to the aforementioned websites for information regarding COVID-19.

### Advertisement design

**Fig. 1:**
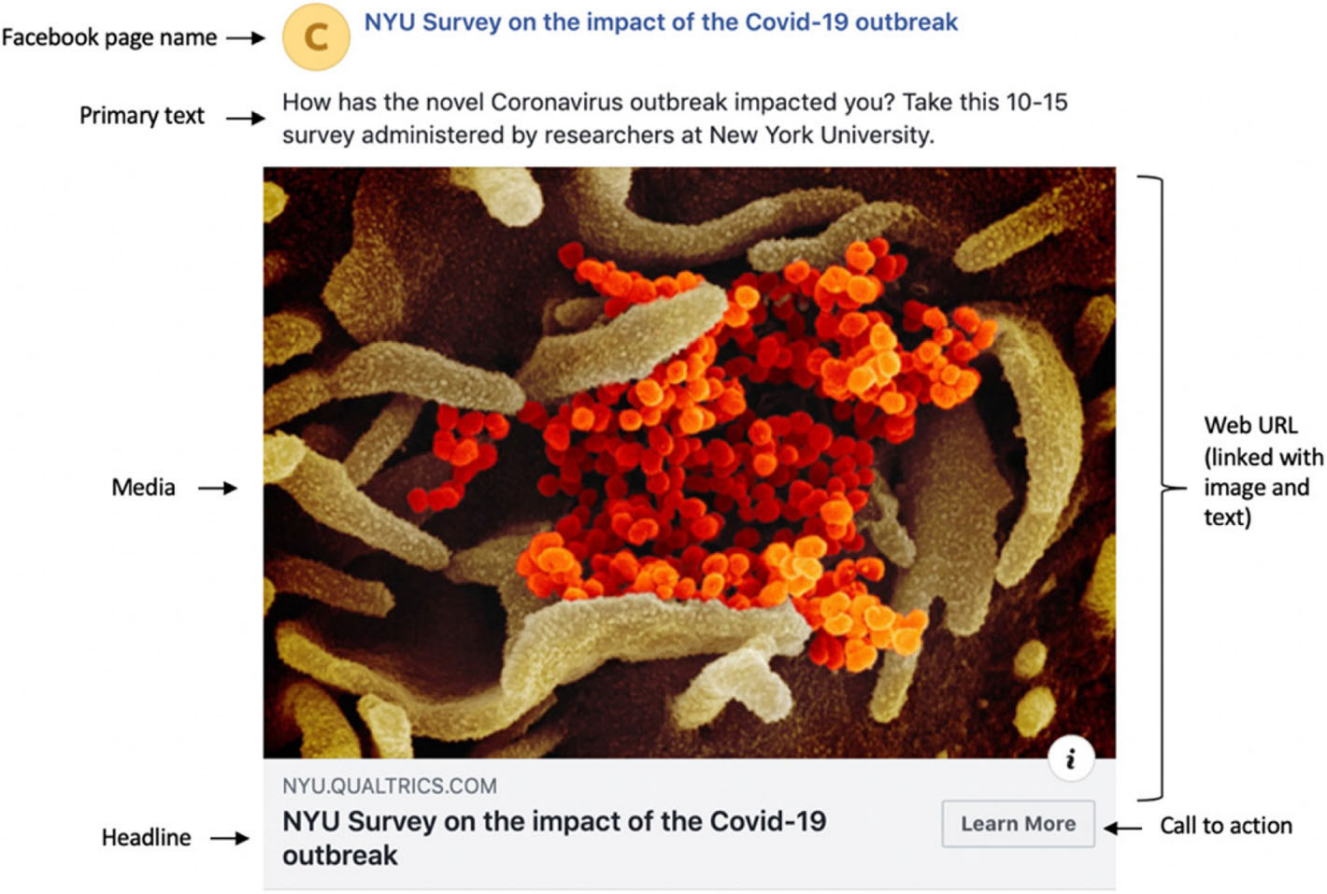
Display of advertisement used for online survey recruitment

A Facebook advertisement campaign was designed to recruit participants for the online survey. The format selected for the advertisement was “Single Image,” which included: 1) media (an image or video); 2) primary text; 3) headline; 4) description; 5) website URL; 6) display link, and; 7) call to action. All the formatting choices except primary text and website URL were optional. To publish the advertisement, a Facebook page was created with the same name as the advertisement headline. As an example, Fig. 1 displays the location of the key components of the advertisement on the Facebook Desktop platform. An image of the SARS-CoV-2 virus published by the National Institute of Allergy and Infectious Diseases Rocky Mountain Laboratories (NIAID-RML) was displayed in the advertisement [23]. The specific locations of each component of the advertisement may have differed based on the platform in which it was placed, with some components (such as the description) only appearing in limited placements. The platforms (described in detail in the following section) included Facebook, Instagram, Messenger, and the Facebook Audience Network. All study materials and procedures, including questionnaire and advertisement materials, underwent Human Subjects Research review. The study was determined exempt by the New York University Institutional Review Board.

Although the entirety of the recruitment campaign ultimately occurred on Facebook (and affiliated social media platforms), the initial recruitment strategy also included the development of a Google advertisement with similar content. However, after attempting to launch the Google advertisement on March 19, 2020, the campaign was terminated after approximately 3 days after failing to achieve any impressions (views of the ad). The specific reasons for the difficulties with the Google advertisement remained unclear. Therefore, we are unable to make any conclusions regarding the applicability of this platform for COVID-19 related research.

### Recruitment strategy

Although a budget of approximately $1000 was and a maximum recruitment time from of approximately one month predetermined, both the budget and time of the recruitment campaign were kept flexible due to uncertainties on cost per click and how many responses per day could be achieved. The marketing objective set for the advertisement was “traffic” (for advertisements aimed at sending people to a particular destination, such as a website designated for data collection) within the broader “consideration” marketing category. Audience targeting was defined as: 1) individuals residing in the United States; 2) ages 18+ years; and; 3) all genders. Although Puerto Rico was included as an option for place of residence within the Qualtrics Survey, the destination setting of the advertisement campaign did not include United States territories. Detailed targeting was turned off for the advertisement.

The “automatic placement” setting was selected for the advertisement, which allowed Facebook’s delivery system to allocate the budget across its multiple affiliated platforms based on where its optimization algorithm estimated the advertisement would perform best. These platforms included Facebook, Instagram, Messenger, and the Facebook Audience Network [24]. Within each platform, examples of where advertisements could be placed included in individual newsfeed, as a “story” (a visual newsfeed), and in-stream (within videos on the platform). The Facebook Audience Network is a group of Facebook-approved mobile apps and websites which allow Facebook advertisements to be shown. According to Facebook, inclusion of the Audience Network in a Facebook advertising campaign can increase success rates of an advertisement by a factor of 8 [25]. Although advertisement placement is driven by factors such as advertisement type, content, and design, the delivery system primarily aims to maximize the number of optimization events (e.g., clicks on an advertisement link) to achieve the lowest average cost per optimization event (e.g., cost per link click) across the various platforms.

In establishing a budget for the advertisement, Facebook requires the selection of an optimization method for advertisement delivery (e.g., maximizing the number of impressions or clicks). The “link clicks” optimization strategy was chosen to cause the delivery algorithm to attempt to show the advertisement to people most likely to click it. An initial daily budget of $20 was set for the first day of advertising. The amount was manually adjusted throughout the recruitment period based on monitoring of the response rate. The daily budget reflected an approximate amount Facebook would spend on distributing the advertisement each day. If the delivery algorithm detected an opportunity to acquire more results on a particular day it could spend up to 25% above the daily budget [26]. However, it would approximately maintain the stipulated total budget across multiple days. Although the delivery strategy was focused on link clicks, the costs of advertisement delivery were based on impressions, incurring a cost each time the advertisement was shown. Payment based on link clicks was only available for advertisement accounts with several weeks of history consistently following Facebook policies, and this option was therefore unavailable for this study.

### Recruitment adjustments

By the sixth day of recruitment it was observed that disproportionately more women were being shown the advertisement than men despite no changes in stipulated targeting, as reflected in the proportion of participants that were male declining progressively from 38 to 27%. This drop in male reach was likely a reflection of the smaller male response rate (number of clicks over number reached), resulting in a greater cost per click for men, which may have triggered the Facebook algorithm to target more women to increase the efficiency of advertisement delivery. Although the significance of this gender bias and the need for interventions had not been pre-planned, to address this issue, a supplemental advertisement (Ad2) was created near the end of the sixth day with the same settings as the original (Ad1) with the exception that it only targeted men. The male-only setting of Ad2 came into full effect on the seventh day after it was initially approved with the exact same gender settings as Ad1. The daily budget for Ad1 was then decreased while the budget for Ad2 (male targeted ad) was increased for about 4 days of recruitment. Through this adjustment, the cumulative percent of male respondents markedly increased from 20 to 40% by the end of the survey. The creation of a supplemental advertisement (as opposed to adjusting the settings of the initial advertisement to male-only) was to ensure that women and those with non-binary sexes were still able to be recruited throughout the entirety of the campaign, albeit in smaller numbers.

On the tenth day of recruitment, we observed skewing in the racial composition of the sample; significantly more Non-Hispanic white responses per day relative to other ethnic/racial groups, rising from 87.9% on day two to 93.0% on day 9. Although Facebook does not have specific information on the race/ethnicity of its users, advertisements can, nonetheless, be broadly targeted to specific racial/ethnic groups using “multicultural affinity targeting.” This targeting allows the advertisement to be shown to individuals whose online activity suggests interest or alignment with a particular cultural group. Thus, another supplemental advertisement (Ad3) was created with the same settings as original as ad (Ad1) except for the addition of a layer of detailed targeting for multicultural affinity directed towards African Americans, Hispanics, and Asian Americans. However, since Ad3 (unlike Ad2) was implemented on the final day of recruitment, its impact could not be substantively evaluated.

Throughout the recruitment process, Facebook advertisements were monitored regularly, and any comments left in the comments section under the advertisements were removed as soon as possible. This was done to minimize influence on potential respondents’ decision to participate by supportive comments as well as the decision to not participate by disparaging comments. Facebook does not allow comments to be hidden automatically, and as such manual removal was required. The evaluation plan of recruitment data was also predetermined based on the data to be provided by both Facebook (i.e. gender and age distribution of impressions and clicks, cost per clicks) and the Qualtrics survey responses.

## Results

### Recruitment summary

A cumulative total of 236,017 individuals were reached (were shown the ad) across the advertisement campaigns, resulting in a total of 9609 clicks. The cumulative conversion rate (number of clicks per individuals reached) was 4.1%, with a rate of 4.9% among females and 3.5% among males. Daily conversion rates ranged from 3.5 to 4.9%. The cumulative participation rate (number of responses per individuals reached) of the survey was 2.8%.

A total of 6602 participant responses were recorded, of which 6562 (99.4%) met study eligibility criteria. Participants took an average of 12.2 min to complete the survey. A total of 171 eligible participants completed the screening questions but opted not to begin the survey. Of the 6391 eligible participants who began the survey, 4998 (78.3%) completed the full survey. The number of recorded responses ranged from 31 (day 1) to 1072 (day 7) per day, although this depended, to a large extent, on the amount spent each day, which ranged from $18.41 (day 1) to $146.64 (day 7). Of the 1393 partial responses recorded, 169 (12.1%) completed at least 75% of the survey, 488 (35.0%) completed at least 50% of the survey, and 829 (59.5%) completed at least 25% of the survey.

Demographic disparities in survey completion could be assessed using the demographic variables included the start of the study (sex and age). With respect to sex disparities in survey completion, the proportion of males among those who completed the survey (40.3%) was lower than the proportion among those who did not (44.4%). With respect to age disparities, there was a decrease in the proportion of 18–29 year olds (10.6 to 8.9%), 30–39 year olds (18.2 to 15.7%), and 80+ year olds (1.1 to 0.4%) among completed responses compared to those who did not complete, an increase among 50–59 year olds (26.3 to 27.9%) and 60–69 year olds (19.5 to 22.7%), while relatively no change among 40–49 and 70–79 year olds.

### Impact of recruitment adjustment

**Fig. 2:**
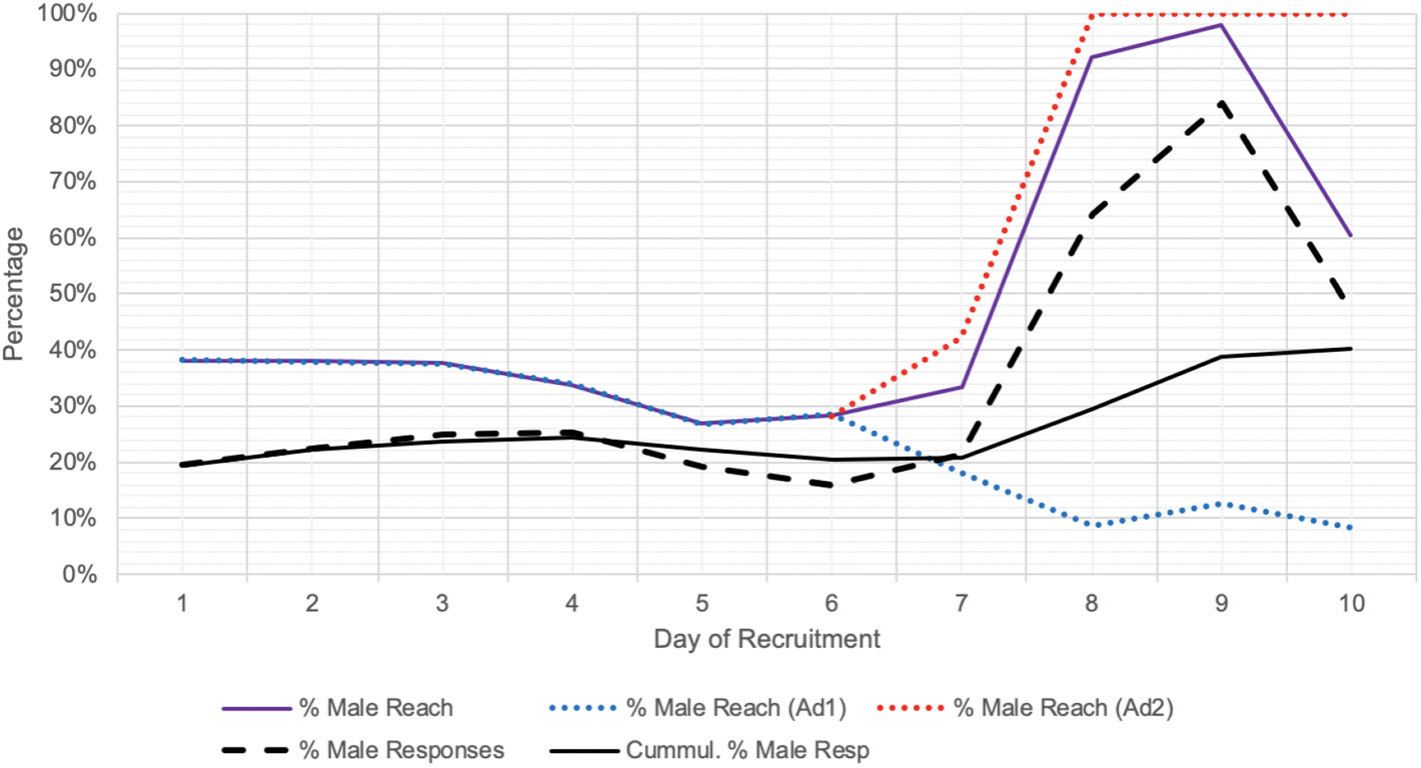
Effect of supplementary male-only advertisement (Ad2) on improving male reach. % Male Reach: daily number of men reached per daily total number reached; % Male responses: daily number of self-identified male responses per daily total responses

**Fig. 3:**
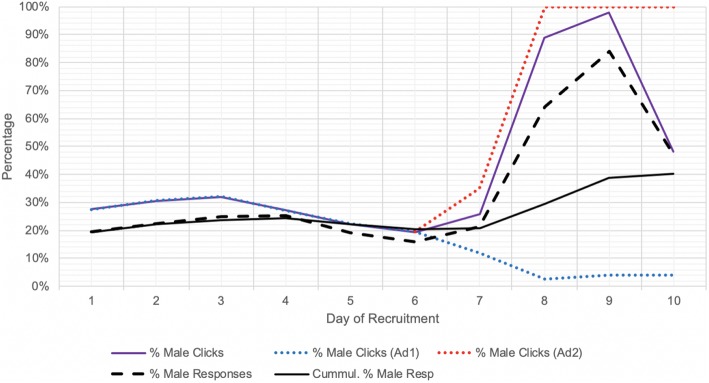
Effect of supplementary male-only advertisement (Ad2) on improving male click. % Male Clicks: daily number of men clicking on the ad per daily total number of clicks; % Male responses: daily number of self-identified male responses per daily total responses

The implementation of a supplemental male-only advertisement (Ad2) markedly enhanced the cumulative percentage of male respondents. This improvement was both reflected by an increased percentage of male social media users reached (Fig. 2) and the subsequent increase in percentage of male social media users clicking on the advertisement (Fig. 3). Ad2 was implemented on day 6, although the male-only targeting was implemented on day 7, prior to which Ad2 had identical settings to Ad1. On days 8 and 9, 95.1% of the average daily budget was spent on Ad2 as opposed to Ad1, although this decreased to 55.1% on day 10.

### Cost analysis

A total of $906 was spent over the recruitment period. The first day of implementation displayed the highest cost per click ($0.17) and cost per response ($0.59). However, costs were relatively stable, with an average cost per click of $0.09 (daily rate of $0.07 to $0.17), an average cost per response of $0.14 (daily rate of $0.11 to $0.59), and an average cost per full response of $0.18 (daily rate of $0.13 to $0.59). The true cost per eligible participant (excluding both those who did not meet eligibility screening criteria and eligible participants who did not begin the survey) was $0.14. Across the first 5 days of recruitment with Ad1, the daily cost per click by gender began at $0.15 for women and $0.21 for men, although by the fifth day the daily cost per click dropped to $0.08 for women and $0.09 for men. On the dates during which Ad2 exclusively recruited men, the cost per click for Ad2 was $0.11.

### Participant characteristics

Of the total 6602 respondents, 40 did not meet study eligibility screening criteria and 171 eligible participants opted not to begin the survey, yielding a final sample size of 6391. An overview of participant characteristics is displayed in Table 1.

**Table 1:** Demographic characteristics of participants in the COVID-19 online survey, *n* = 6391

Variable	Total, n (%)
**Sex**	
Female	3688 (57.7)
Male	2633 (41.2)
Other/No disclosure	70 (1.1)
**Age group (years)**	
18–29	595 (9.3)
30–39	1038 (16.2)
40–49	1177 (18.4)
50–59	1758 (27.5)
60–69	1409 (22.0)
70–79	381 (6.0)
80+	33 (0.5)
Race/ethnicity*	
Non-Hispanic Black	37 (0.7)
Non-Hispanic White	4613 (92.3)
Asian/Pacific Islander	51 (1.0)
Hispanic/Latinx	145 (2.9)
Native American or American Indian	50 (1.0)
Interracial, Mixed race, or Other	102 (2.0)
Region*	
Northeast	1278 (25.6)
Midwest	1490 (29.8)
South	1475 (29.5)
West	754 (15.1)
Puerto Rico	1 (0.02)
Residence type*	
Rural	1710 (34.2)
Suburban	2433 (48.7)
Urban	855 (17.1)
Employment status*	
Employed	2736 (54.7)
Unpaid work (e.g., homemaker, eldercare, childcare)	209 (4.2)
Self-employed	403 (8.1)
Out of work and looking for work	156 (3.1)
Out of work but not currently looking for work	155 (3.1)
Student	126 (2.5)
Retired	957 (19.1)
Unable to work	256 (5.1)
Work in healthcare/clinical setting*	
Yes	772 (15.4)
No	4226 (84.6)
Children under 18*	
Yes	1545 (30.9)
No	3453 (69.1)
Educational attainment*	
High School or below	785 (15.7)
Some college	1711 (34.2)
Bachelor’s degree	1376 (27.5)
Masters/Professional degree or above	1126 (22.5)
**Political affiliation**	
Democrat	1684 (33.7)
Republican	1282 (25.7)
Other	940 (18.8)
Prefer not to disclose	1092 (21.8)

*n = 4998 as *n* = 1393 (21.8%) respondents submitted incomplete surveys

Percentages presented excluded participants with missing responses from denominators. Percentages may not sum to 100 due to rounding

Participants from diverse geographic, employment, and educational backgrounds were able to be recruited. Responses were recorded from all 50 U.S. states and the District of Columbia and Puerto Rico, with an average of 96.1 responses per state (interquartile range of 35.8 and 121.5). A majority of the participants self-identified as Non-Hispanic White (*n* = 4613, 92.3%). Age and educational attainment were well distributed, with the largest groups being those aged 50 to 59 years (*n* = 1758, 27.5%) and those with some college attainment (*n* = 1711, 34.2%). Political affiliation among the two major political parties was comparably distributed; Democrats (*n* = 1684, 26.4%) and Republicans (*n* = 1282, 20.1%). Responses from rural areas (*n* = 1710, 34.2%) were greater than urban areas (*n* = 855, 17.1%), although approximately half of the participants resided in suburban areas (*n* = 2433, 48.7%).

## Discussion

### Results

The findings from this study support the utility of social media platforms as a tool to efficiently and effectively recruit large and diverse samples of survey respondents, especially during rapidly evolving global or regional health crises such as COVID-19 when other methods of recruitment are no longer safe, practical, economically feasible, or even legal. The success of recruitment in this survey supports evidence from a recent systematic review which identified Facebook’s usefulness for reducing costs, shortening recruitment periods, and enhancing representativeness of target populations [4]. However, most past studies involving Facebook-based recruitment have specifically targeted certain subsets of a population based on behavioral or demographic outcomes (and often those this platform for recruitment based on these specific characteristics), which significantly different from the current study’s goal to gain a broadly representative national sample [4]. Indeed, there were obvious limitations in this method’s ability to ensure sample representativeness across all sociodemographic variables, monitoring recruitment of ethnic/racial groups and men, in this case, and over-sampling of participants with specific sociodemographic characteristics through implementation of compensatory advertisements may, at least partly, circumvent some of these limitations.

In interpreting the representativeness of the population surveyed, key demographic characteristics were compared with 2018 estimates from the U.S. Census Bureau [27–30] (Table 2). Overall, the data was relatively representative of the U.S. age distribution, although with a comparatively lower proportion of younger adults [27]. The gender composition of the sample was comparable to the US population (56.2% female in the study compared to 51.0% across the nation) [27], although this was attributable to the supplementary male-only advertising implemented during recruitment. However, the composition of Non-Hispanic whites in the study was significantly higher than in the U.S. (92.3% compared to 60.5%) [31]. The overrepresentation of white respondents in social media-based research studies has been well documented and represents a significant limitation of the platform [4, 5]. Educational attainment, likewise, displayed mixed representativeness; those with some college attainment were overrepresented (34.2% compared to 18.0%), although this may be in part because the U.S. Census defined the category as “some college, no degree” while the “no degree” component was not defined in the current study [29]. Representativeness of employment distribution was difficult to assess due to differences in terminologies used in the study with the U.S. Census categories. These limitations are in part due to the fact that the questionnaire items for the demographic characteristics were informed by past surveys rather than directly from the U.S. Census categories. In addition, the fact that the recruitment period corresponded with a time of dramatic increase in unemployment [32] may have impacted the data distribution.

**Table 2:** Comparison of study sample characteristics with 2018–2019 U.S. Census estimates

Variable	Percentage of study sample	Percentage of adult U.S. population
**Sex**		
Female	57.7	51.3
Male	41.2	48.7
**Age group (years)**		
18–29	9.3	21.3
30–39	16.2	17.2
40–49	18.4	15.9
50–59	27.5	16.9
60–69	22.0	14.7
70–79	6.0	8.9
80+	0.5	5.0
**Race/ethnicity**		
Non-Hispanic Black	0.7	13.4
Non-Hispanic White	92.3	60.4
Asian/Pacific Islander	1.0	6.1
Hispanic/Latinx	2.9	18.3
Native American or American Indian	1.0	1.3
Interracial, Mixed race, or Other	2.0	2.7
**Region**		
Northeast	25.6	17.1
Midwest	29.8	20.8
South	29.5	38.3
West	15.1	23.9
**Educational attainment**		
High School or below	15.7	39.5
Some college	34.2	18.5
Bachelor’s degree	27.5	20.6
Masters/Professional degree or above	22.5	11.6

Percentages may not sum to 100 due to rounding. Census estimates for age, sex, and education are among adults aged 18 years and older, while estimates for race and region are among the total population

### Limitations

The reliance on a social media platform that operates via mechanisms that were largely out of the control of study investigators imposed a number of important limitations. We intend to conduct additional waves of data collection using a similar survey methodology to track temporal changes in public perceptions and behaviors as the COVID pandemic progresses. Lessons learned from the acknowledgement of these limitations will serve to improve future surveys by us as well as other investigators. First, the over-representation of Non-Hispanic white women and the corresponding under-representation of men and ethnic/racial minorities, compromised the representativeness of the sample and, consequently, the extent to which findings may be extrapolated to the general U.S. population. A recent in-depth analysis of the Facebook advertisement delivery optimization algorithm suggests that the platform uses complex automated advertisement content analyses to steer advertisements towards certain subsets of its users (e.g., men or women) [33]. Such insights suggest that the content or preliminary data from the COVID-19 survey advertisement may have been interpreted by the underlying algorithm to display the advertisement to more women. Nonetheless, the attempt to ameliorate this skewing by creating a supplementary male-only advertisement was effective in enhancing total and percent male reach and responses, suggesting that any unforeseen skewing incurred from the Facebook advertisement delivery algorithm can be addressed by such measures.

Third, although all advertisements passed through the Facebook review process and were implemented successfully, some were disabled or re-enabled throughout the study period. Ad1 was temporarily disabled on the fifth day of recruitment citing article 27 of the Facebook Advertising Policies on Unacceptable Business Practices; however, following an appeal the advertisement was quickly re-enabled and actively recruited participants until the end of the study period. However, during the eleventh and final day of recruitment Ad2 was similarly disabled for the same reason, although upon similar appeal, it was not re-enabled (despite having identical content to the re-enabled Ad1). Although few past studies using Facebook or other social media platforms have explicitly documented issues of disabled advertisements or advertisements failing to pass through review [5], specifics of advertisement imagery have been cited as barriers in the Facebook review process [34]. However, this obstacle may also be explained by an announcement posted by Facebook during the recruitment period that, due to the impact of the COVID-19 pandemic on Facebook and the need to relocate contract workers in charge of content and advertisement review, it would be reducing the number of appeals getting reviewed (as well as longer response times for ongoing appeals) [35]. Such a barrier may also be significant for future studies attempting to use Facebook-based recruitment for COVID-19 research specifically, as well as other research in times of large scale and disruptive health crises.

### Comparison with prior work

Although survey research focused on the COVID-19 pandemic is in its early stages, a similar cross-sectional Qualtrics-based COVID-19 online survey using the Prolific online platform (a website which connects researchers with individuals interested in participating in online studies) recruited 5974 participants across two rounds of 2–3 day recruitment periods between February 23 and March 2, 2020 [2]. Indeed, while a similar number of participants were recruited to the study in a shorter time period, participants were paid $1.50 for completing the questionnaire [2] (8.3 times higher than the $0.18 average cost per full response in the current study), suggesting a strength of social-media based recruitment in minimizing cost per response in the context of COVID-19 and potentially in-progress health crises in general.

The desirability of social media-based recruitment for COVID-19-related research is also supported by a similar online COVID-19 knowledge, attitudes, and practices survey conducted in China among 6910 individuals [3]. The survey similarly utilized social media platforms (notably WeChat and Weibo) to recruit participants between January 27 and February 1, 2020 (approximately 6 days) [3]. However, the study authors largely relied upon personal networks of people living in Hubei province, and also posted the advertisement to a select number of institutional WeChat accounts and local popular media outlets, which may have resulted in the fact that 55.1% of participants resided in Hubei province alone [3]. Indeed, the relatively diverse and well-distributed geographic distribution across the sample was facilitated by using advertisements on social media in the current study and suggest another key strength of this specific use of the platform to obtain national samples on COVID-19 or potentially other rapidly evolving health crises. A further strength this social media advertisement campaign was observed to provide was the ability to effectively monitor and intervene on observed gender biases to enhance the representative of the study sample.

Finally, while the experience of using Facebook-based participant recruitment for COVID-19 research largely mirrored past studies using this platform, there were some key differences. Aside from the aforementioned similarities in experiencing over-representation of white and female populations, the over-representation of individuals with higher education is also a salient issue [4]. Likewise, the 4.1% conversation rate (which was link clicks per individuals reached in this context) matched the 4% average observed in past studies [4]. However, the recruitment period of 11 days was significantly shorter than 5.13-month average in Facebook-based health research. Moreover, the average cost per click ($0.09) and cost per eligible participant ($0.14) was markedly lower than the health research average of $0.57 and $19.71 [4]. The greater cost-effectiveness of utilizing Facebook for COVID-19 research may be reflection of conducting a recruitment campaign during a period of great public interest on the topic, highlighting that collecting large quantities of survey data during ongoing global health crises may be particularly suited for social media-based campaigns.

## Conclusion

Facebook, as a social media platform, can be an effective and cost-efficient research strategy to collect valuable, large-scale, nationwide survey data on ongoing pandemics such as COVID-19; with the caveat that close monitoring of responses rates of various sociodemographic subgroups is necessary, and may require implementation of appropriate supplemental strategies to mitigate these challenges. Although among survey respondents the proportion of males who completed the survey was lower than the proportion among those who did not, the interventions conducted during the recruitment period were able to successfully increase the overall number of male responses, and this was able to significantly enhance the representative of the sample in comparison to U.S. Census statistics. Researchers should acknowledge any obstacles placed by advertisement delivery optimization algorithms or the realities of advertisement review challenges during times of global health crises. Although the current study was unable to obtain substantive data on the effectiveness of “multicultural affinity” targeting to enhance sampling of racial or ethnic minority population, future research on COVID-19 or other global health crises using social media campaigns should also evaluate such measures. However, creating supplementary gender-based targeting has the potential to appropriately enhance response rates if certain gender-based groups (such as men) are being progressively under-sampled by advertisement campaigns. Notwithstanding the aforementioned challenges and caveats, in a rapidly emerging health crisis, such as the COVID-19 pandemic, a social media campaign has the capacity to capture much needed empirical data that may be valuable for the planning, development, and implementation of health communication campaigns to mitigate health threats.

## Supplementary information

**Additional file 1.**

## Data Availability

The datasets used and/or analyzed during the current study are available from the corresponding author on reasonable request.

## Abbreviations

U.S.: United States
MERS: Middle East respiratory syndrome
SARS: Severe acute respiratory syndrome
Ad1: Advertisement 1
Ad2: Advertisement 2
Ad3: Advertisement 3
