# Supplementary material for: Social media as a recruitment platform for a nationwide online survey of COVID-19 knowledge, beliefs, and practices in the United States: methodology and feasibility analysis
Source: BMC Med Res Methodol. 2020 May 13;20:116. doi: 10.1186/s12874-020-01011-0 (PMC7220591; doi:10.1186/s12874-020-01011-0)
Supplement: Supplementary file 1 — Additional file 1. [file 12874_2020_1011_MOESM1_ESM.docx]

NYU Survey on the impact of the Covid-19 outbreak

**Screening questions**

Are you 18 or older?

1. Yes
2. No

Do you reside in the US?

1. Yes
2. No

[If answered No to any of these questions, then END OF SURVEY]:

**1.0 Preliminary Demographics + Travel Questions**

1.1 Sex

1. Female
2. Male
3. Other
4. Prefer not to disclose

1.2 Age group

1. 18-29 years old
2. 30-39 years old
3. 40-49 years old
4. 50-59 years old
5. 60-69 years old
6. 70-79 years old
7. 80+ years old

1.3 How many international trips by plane have you taken since December 1, 2019?

1. 0
2. 1-2
3. 3-4
4. 5-6
5. 7 or more

1.4 How many domestic trips by plane have you taken since December 1, 2019?

1. 0
2. 1-2
3. 3-4
4. 5-6
5. 7 or more

1.5 I believe my risk of catching Coronavirus by flying is…

1. Extremely Low
2. Low
3. Moderate
4. High
5. Extremely High

**2.0 Knowledge**

*Below are a series of statements about Coronavirus. Please read each statement and indicate if you believe each statement is either true or false.*

2.1 Coronavirus is a contagious disease.

1. True
2. False

2.2 A person infected with Coronavirus is not contagious until after symptoms appear.

1. True
2. False

2.3 Coronavirus cannot be spread through sneezing and coughing.

1. True
2. False

2.4 Currently, there is an FDA approved drug for treating individuals with Coronavirus.

1. True
2. False

2.5 Coronavirus can live on surfaces outside of the body for a few hours or several days.

1. True
2. False

2.6 There is no vaccine currently available to prevent infection with Coronavirus.

1. True
2. False

2.7 Children are at high risk for complications from Coronavirus.

1. True
2. False

2.8 Older people with other health conditions are more likely to die from Coronavirus.

1. True
2. False

2.9 People with Coronavirus can have no symptoms at all.

1. True
2. False

2.10 Most people with Coronavirus will have severe or critical symptoms.

1. True
2. False

2.11 Alcohol-based hand sanitizers cannot protect you from Coronavirus.

1. True
2. False

2.12 Coronavirus may be transmitted by mosquito bites.

1. True
2. False

2.13 Coronavirus originated from animals.

1. True
2. False

*How can you protect yourself from being* ***INFECTED*** *with Coronavirus?*

2.14 Washing your hands frequently with soap and water.

1. True
2. False

2.15 Getting a flu shot.

1. True
2. False

2.16 Wearing a face mask.

1. True
2. False

2.17 Stop going to school/work.

1. True
2. False

2.18 Wiping potentially contaminated surfaces with a disinfectant.

1. True
2. False

2.19 Staying away from Asian people.

1. True
2. False

2.20 Staying away from people who sneeze and cough.

1. True
2. False

2.21 Avoiding touching your eyes, nose and mouth.

1. True
2. False

2.22 Taking antibiotic.

1. True
2. False

2.23 Stop eating Chinese food.

1. True
2. False

**3.0 Behavior and Information Sources**

3.1 In the last week, how often have you searched for information about Coronavirus?

1. Multiple times a day
2. Once a day
3. A couple of times a week
4. Once a week
5. Never

*Do you get information about Coronavirus from any of the below sources?*

3.2 Spouse/Partner.

1. Yes
2. No
3. Not applicable

3.3 Other family members.

1. Yes
2. No
3. Not applicable

3.4 Friends or Coworkers.

1. Yes
2. No
3. Not applicable

3.5 Religious leader (e.g., minister).

1. Yes
2. No
3. Not applicable

3.6 Doctor/medical provider.

1. Yes
2. No
3. Not applicable

3.7 TV.

1. Yes
2. No
3. Not applicable

3.8 Radio or Podcasts.

1. Yes
2. No
3. Not applicable

3.9 Newspaper (printed or internet, e.g., the New York Times).

1. Yes
2. No
3. Not applicable

3.10 Government or other official websites (e.g., the CDC or WHO).

1. Yes
2. No
3. Not applicable

3.11 Social media (e.g., Facebook or Twitter).

1. Yes
2. No
3. Not applicable

3.12 Google search, Wikipedia or other non-government websites.

1. Yes
2. No
3. Not applicable

3.13 Do you seek information on Coronavirus from mainstream media sources (e.g., CNN, Fox News, MSNBC, Local/national networks, International networks)?

1. Yes
2. No

3.14 [If YES to 3.13] Which of the below mainstream media sources do you get the most information from?

1. CNN
2. Fox news
3. MSNBC
4. Other local or national networks
5. Other International networks (e.g., BBC, Al Jazeera, Sky News)

3.15 Which information source below do you trust the most for Coronavirus (select one)?

1. Spouse/Partner
2. Other family members
3. Friends or Coworkers
4. Religious leader (e.g., minister)
5. Doctor/medical provider
6. TV
7. Radio or Podcasts
8. Newspaper (printed or internet, e.g., the New York Times)
9. Government or other official websites (e.g., the CDC or WHO)
10. Social media (e.g., Facebook or Twitter)
11. Google search, Wikipedia or other non-government websites

3.16 I believe the information I get about Coronavirus is accurate.

1. Yes
2. No

3.17 I believe the information I get about Coronavirus is up to date.

1. Yes
2. No

*To protect myself from getting* ***INFECTED*** *with Coronavirus, I ...*

3.18 Got a flu shot (or had my children get a flu shot) after hearing about Coronavirus.

1. Yes
2. No

3.19 Purchased a face mask.

1. Yes
2. No

3.20 Started working from home.

1. Yes
2. No

3.21 Started using hand-sanitizer and/or washing my hands more often.

1. Yes
2. No

3.22 Started drinking more fluids and/or getting more rest .

1. Yes
2. No

3.23 Started taking antiviral and/or antibiotics.

1. Yes
2. No

3.24 Started taking dietary supplements (e.g., vitamins, probiotics).

1. Yes
2. No

3.25 Avoided using public transportation.

1. Yes
2. No

3.26 Kept away from crowded places.

1. Yes
2. No

3.27 Started cleaning and/or disinfecting things that I might touch (e.g., doorknobs, phone).

1. Yes
2. No

3.28 Started wearing rubber gloves in public.

1. Yes
2. No

3.29 Started taking more hot baths .

1. Yes
2. No

*In the past seven days, because of Coronavirus I have …*

3.30 Maintained a stockpile of food and/or water.

1. Yes
2. No

3.31 Cancelled/postponed a social event (e.g., meeting friends, eating out, going to sports events).

1. Yes
2. No

3.32 Discussed with a friend or family member what we would do if one of us catches coronavirus.

1. Yes
2. No

3.33 Stayed away from individuals at risk for severe Coronavirus (e.g., the elderly).

1. Yes
2. No

*Since hearing about the Coronavirus outbreak, how have the below behaviors changed for you?*

3.34 Smoking (tobacco products)

1. Much more
2. Little more
3. Not changed
4. Little less
5. Much less
6. Not applicable

3.35 Drinking (alcohol)

1. Much more
2. Little more
3. Not changed
4. Little less
5. Much less
6. Not applicable

3.36 Sleeping

1. Much more
2. Little more
3. Not changed
4. Little less
5. Much less
6. Not applicable

*Until the Coronavirus outbreak is controlled I would* ***not****:*

3.37 Travel to any of the countries severely affected by Coronavirus.

1. Strongly Agree
2. Agree
3. Disagree
4. Strongly Disagree

3.38 Take a cruise.

1. Much more
2. Little more
3. Not changed
4. Little less

3.39 Travel by plane internationally.

1. Much more
2. Little more
3. Not changed
4. Little less

3.40 Travel by plane within the United States.

1. Much more
2. Little more
3. Not changed
4. Little less

**4.0 Beliefs and Risk Perceptions**

4.1 What do you think is your risk of getting infected with the Coronavirus?

Not at all likely Extremely likely

0 1 2 3 4 5 6 7 8 9 10

4.2 In the **NEXT 3 MONTHS**, how many people in the United States do you think will contract the Coronavirus?

1. Up to 100
2. Up to 1,000
3. Up to 10,000
4. Up to 100,000
5. Up to 1,000,000
6. Up to 10,000,000
7. More than 10,000,000

4.3 If you were infected with the Coronavirus, how severe do you think it would be?

Not Severe Very Severe

0 1 2 3 4 5 6 7 8 9 10

*Please read each statement and indicate your level of agreement.*

4.4 If I were ORDERED to quarantine myself due to Coronavirus, I would do so.

1. Strongly Agree
2. Agree
3. Disagree
4. Strongly Disagree

4.5 If I were asked to self-quarantine due to Coronavirus, I would do so.

1. Strongly Agree
2. Agree
3. Disagree
4. Strongly Disagree

4.6 I can financially afford to self-quarantine.

1. Strongly Agree
2. Agree
3. Disagree
4. Strongly Disagree

4.7 Since the Coronavirus outbreak, I feel discriminated against.

1. Strongly Agree
2. Agree
3. Disagree
4. Strongly Disagree

4.8 Since the Coronavirus outbreak, others are staying away from me.

1. Strongly Agree
2. Agree
3. Disagree
4. Strongly Disagree

4.9 I think that Coronavirus was released as an act of bioterrorism.

1. Strongly Agree
2. Agree
3. Disagree
4. Strongly Disagree

4.10 Coronavirus is more deadly than the seasonal flu.

1. Strongly Agree
2. Agree
3. Disagree
4. Strongly Disagree

4.11 The amount of media attention devoted to Coronavirus has been adequate.

1. Strongly Agree
2. Agree
3. Disagree
4. Strongly Disagree

4.12 Coronavirus is not as big of a problem as the media suggests.

1. Strongly Agree
2. Agree
3. Disagree
4. Strongly Disagree

4.13 Coronavirus is a bigger problem than the government suggests.

1. Strongly Agree
2. Agree
3. Disagree
4. Strongly Disagree

4.14 I think warmer weather will reduce the spread of the Coronavirus.

1. Strongly Agree
2. Agree
3. Disagree
4. Strongly Disagree

4.15 I’m more aware of my race/ethnicity when I’m in the public due to the Coronavirus outbreak.

1. Strongly Agree
2. Agree
3. Disagree
4. Strongly Disagree

**5.0 National threat/policy beliefs**

5.1 How prepared is the U.S. federal government to handle the Coronavirus outbreak?

Totally unprepared Extremely prepared

0 1 2 3 4 5 6 7 8 9 10

5.2 How prepared is my state government to handle the Coronavirus outbreak?

Totally unprepared Extremely prepared

0 1 2 3 4 5 6 7 8 9 10

*Please read each statement and indicate your level of agreement.*

5.3 The Coronavirus outbreak is a threat to our national security.

1. Strongly Agree
2. Agree
3. Disagree
4. Strongly Disagree

5.4 The Coronavirus outbreak is a threat to our national economy.

1. Strongly Agree
2. Agree
3. Disagree
4. Strongly Disagree

5.5 The Coronavirus outbreak is a threat to my personal finances.

1. Strongly Agree
2. Agree
3. Disagree
4. Strongly Disagree

5.6 The United States should assume worldwide leadership in confronting the Coronavirus outbreak.

1. Strongly Agree
2. Agree
3. Disagree
4. Strongly Disagree

5.7 The United States should assist other countries in confronting the Coronavirus outbreak by providing financial/medical resources.

1. Strongly Agree
2. Agree
3. Disagree
4. Strongly Disagree

5.8 The United States should increase domestic funding for Coronavirus related medical resources and research.

1. Strongly Agree
2. Agree
3. Disagree
4. Strongly Disagree

5.9 People who travel to countries where the Coronavirus has been detected should be tested for the Coronavirus before being allowed to return to the United States.

1. Strongly Agree
2. Agree
3. Disagree
4. Strongly Disagree

5.10 People who travel to countries where the Coronavirus has been detected should be asked to quarantine for 14 days in the United States, even if they test negative for the Coronavirus.

1. Strongly Agree
2. Agree
3. Disagree
4. Strongly Disagree

5.11 The United States should ban the entry of individuals arriving from countries highly affected by the Coronavirus.

1. Strongly Agree
2. Agree
3. Disagree
4. Strongly Disagree

5.12 The United States should ban the entry of any individual arriving from a foreign country until the Coronavirus outbreak is controlled.

1. Strongly Agree
2. Agree
3. Disagree
4. Strongly Disagree

5.13 I am optimistic that the Coronavirus outbreak will be controlled in the next 3 months.

1. Strongly Agree
2. Agree
3. Disagree
4. Strongly Disagree

5.14 Are you eligible to vote in local, state, or federal elections in the United States?

1. Yes
2. No

*[If YES to 5.14 display 5.15-5.18]*

5.15 Will you consider candidates’ policies on the Coronavirus outbreak when voting in the 2020 elections?

1. Yes
2. No

5.16 Do you think the Coronavirus outbreak will influence who gets elected in the 2020 elections?

1. Yes
2. No

5.17 Will you skip in-person voting if Coronavirus is spreading in your community?

1. Yes
2. No

5.18 Do you think that all people should be allowed to vote by mail (i.e., not in-person voting) if the Coronavirus is spreading in the United States?

1. Yes
2. No

**6.0 Anxiety-Depression (PHQ4) for coronavirus**

*Over the last 7 days, how often have you been bothered by any of the following problems because of THE CORONAVIRUS OUTBREAK?*

6.1 Feeling nervous, anxious, or on edge?

1. Not at all
2. Several days
3. More than half the days
4. Nearly everyday

6.2 Not being able to stop or control worrying?

1. Not at all
2. Several days
3. More than half the days
4. Nearly everyday

6.3 Feeling down, depressed, or hopeless?

1. Not at all
2. Several days
3. More than half the days
4. Nearly everyday

6.4 Little interest or pleasure in doing things (that I used to enjoy)?

1. Not at all
2. Several days
3. More than half the days
4. Nearly everyday

**7.0 Impact of Events Scale (IES-6) for coronavirus**

*In the past 7 days …*

7.1 I thought about the Coronavirus when I didn’t mean to

1. Not at all
2. Several days
3. More than half the days
4. Nearly everyday

7.2 I felt watchful or on-guard.

1. Not at all
2. Several days
3. More than half the days
4. Nearly everyday

7.3 Other things kept making me think about the Coronavirus

1. Not at all
2. Several days
3. More than half the days
4. Nearly everyday

7.4 I was aware that I still had a lot of feelings about the Coronavirus, but I didn’t deal with them.

1. Not at all
2. Several days
3. More than half the days
4. Nearly everyday

7.5 I tried not to think about the Coronavirus.

1. Not at all
2. Several days
3. More than half the days
4. Nearly everyday

7.6 I had trouble concentrating.

1. Not at all
2. Several days
3. More than half the days
4. Nearly everyday

**8.0 Demographics**

8.1 What’s your race/ethnicity?

1. Black, Non-Hispanic
2. White, Non-Hispanic
3. Asian/Pacific Islander
4. Hispanic/Latinx
5. Native American or American Indian
6. Interracial, Mixed race, or Other
   1. In which state do you currently reside?

[Drop-down menu of all 50 states, District of Columbia, Puerto Rico]

8.3 How would you describe where you live?

1. Urban
2. Suburban
3. Rural

8.4 What is your current working status?

1. Employed
2. Unpaid work (e.g., homemaker, eldercare, childcare)
3. Self-employed
4. Out of work and looking for work
5. Out of work but not currently looking for work
6. Student
7. Military personnel
8. Retired
9. Unable to work

8.5 Do you work in an environment where you come into contact with sick people in a healthcare or clinical setting (e.g., doctor, nurse, hospital staff, paramedic, etc.)

1. Yes
2. No

8.6 Do children under 18 years old live in your household?

1. Yes
2. No

8.7 What is your highest level of education?

1. Less than a High School diploma
2. High School diploma or GED
3. Some college
4. Bachelor’s degree
5. Masters/Professional degree or above

8.8 What is your political affiliation?

1. Democrat
2. Republican
3. Other
4. Prefer not to say
